# Infrastructural challenges to better health in maternity facilities in rural Kenya: community and healthworker perceptions

**DOI:** 10.1186/s12978-015-0078-8

**Published:** 2015-11-09

**Authors:** Hildah Essendi, Fiifi Amoako Johnson, Nyovani Madise, Zoe Matthews, Jane Falkingham, Abubakr S Bahaj, Patrick James, Luke Blunden

**Affiliations:** Department of Social Statistics and Demography, University of Southampton, SO17 1BJ Southampton, UK; Engineering and the Environment, University of Southampton, SO17 1BJ Southampton, UK

## Abstract

**Background:**

The efforts and commitments to accelerate progress towards the Millennium Development Goals for maternal and newborn health (MDGs 4 and 5) in low and middle income countries have focused primarily on providing key medical interventions at maternity facilities to save the lives of women at the time of childbirth, as well as their babies. However, in most rural communities in sub-Saharan, access to maternal and newborn care services is still limited and even where services are available they often lack the infrastructural prerequisites to function at the very basic level in providing essential routine health care services, let alone emergency care. Lists of essential interventions for normal and complicated childbirth, do not take into account these prerequisites, thus the needs of most health facilities in rural communities are ignored, although there is enough evidence that maternal and newborn deaths continue to remain unacceptably high in these areas.

**Methods:**

This study uses data gathered through qualitative interviews in Kitonyoni and Mwania sub-locations of Makueni County in Eastern Kenya to understand community and provider perceptions of the obstacles faced in providing and accessing maternal and newborn care at health facilities in their localities.

**Results:**

The study finds that the community perceives various challenges, most of which are infrastructural, including lack of electricity, water and poor roads that adversely impact the provision and access to essential life-saving maternal and newborn care services in the two sub-locations.

**Conclusions:**

The findings and recommendations from this study are important for the attention of policy makers and programme managers in order to improve the state of lower-tier health facilities serving rural communities and to strengthen infrastructure with the aim of making basic routine and emergency obstetric and newborn care services more accessible.

## Aim of study

**Fig. 1:**
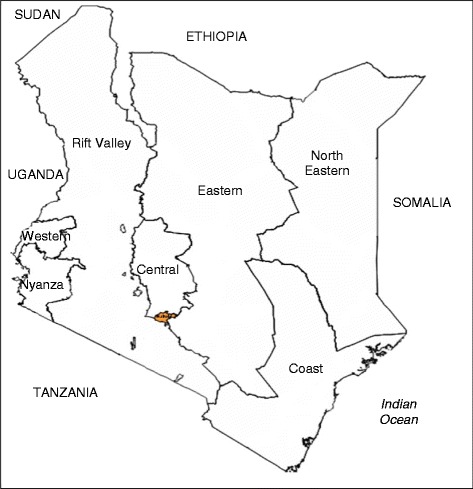
Map showing Kenyan provinces

Kenya is one of the sub-Saharan African countries lagging behind in reaching the fourth and fifth Millennium Development Goals (MDGs). The two most recent (2003 and 2008) Demographic and Health Surveys in the country revealed that the maternal mortality ratio increased from 412 to 488 maternal deaths per 100,000 live births, with the highest proportions recorded in rural communities [1, 2]. A lack of basic infrastructure including quality water and electricity supply has been associated with poor quality health services in rural Kenya [1, 3]. Only 58 % of all hospitals in the country have an all-year supply of water, while one-quarter have uninterrupted electricity supply [4]. Residents in Eastern province face the most severe shortfalls in basic infrastructure; 40 % of facilities do not have either an uninterrupted electricity supply or a generator with fuel, and not surprisingly, only 42.8 % of women access a skilled attendant at childbirth, often citing long distances to a health facility as a major deterrent [5]. (See Fig. 1 for map of Kenyan provinces).

Despite the clear lack of essential amenities needed to provide very basic health services in rural Kenya, especially in Eastern province [3], there has been little research to understand the challenges faced by health providers and service users in the provision and access of this care and their perceptions of key infrastructural barriers.

In this study, we use qualitative data collected using key informant interviews and Focus Group Discussions (FGDs) to explore how community members and their health providers perceive the challenges faced in accessing and providing essential maternal and newborn care services, the coping mechanisms adopted by both providers and service users and their effect on quality of care in two rural communities (Kitonyoni and Mwania) in Eastern Kenya.

## Background and rationale for study

It is almost three decades since the launch of the Safe Motherhood Initiative in 1987 when policy-makers declared their intentions and commitments to reduce the high levels of maternal and newborn mortality in developing countries. But, maternal and newborn deaths remain unacceptably high, particularly in sub-Saharan Africa where more than one-half (56 %) of all maternal deaths continue to occur [6] and more than 1,000 000 children die within the first month of birth [7] with rural communities bearing the highest burden [6, 7]. In these communities, poor women are confronted with infrastructural challenges, impeding access to health care and consequently, compromising health outcomes [8–11].

Countries with high maternal and newborn deaths need to invest in universal access to essential Emergency Obstetric and Newborn Care (EmONC) services. that can deliver lifesaving services and interventions facilities [12, 13, 14]. The functionality of health facilities providing maternal and newborn care has been assessed using a set of nine key life-saving interventions often referred to as “signal functions” [15, 16]. Lower-tier health facilities in rural areas require only seven signal functions to be working to qualify as ‘functioning’, but all of these functions require multiple infrastructural support to be operational. Many lack electricity, water, adequate health personnel and in general, rural areas have poor roads that act as barriers in the access and referrals for maternal and newborn care [6, 17–20]. (See Table 1 for the signal functions and their infrastructural requirements).

**Table 1:** The nine signal functions for maternity facilities and their infrastrutural needs

Signal function	Required in Basic or Comp-rehensive facilities?	Infrastructural needs				
		Electricity	Clean water	Human resources	Enabling medical environment (drugs/supplies/equipment)	Road/vehicular access
1. Administer parenteral antibiotics for maternal infection	B and C	Lighting		HW with midwifery competencies	Drugs	Yes
		Lab tests				
2. Administer uterotonic drugs for haemorrhage	B and C	Lighting	Clean blood spills	HW with midwifery competencies	Drugs	Yes
					Supplies	
3. Administer parenteral anticonvulsants for pre-eclampsia and eclampsia (e.g. magnesium sulphate)	B and C	Lighting		HW with midwifery competencies	Drugs	Yes
		Lab tests			Lab equipment	
4. Perform manual removal of placenta for retained placenta	B and C	Lighting	Wash hands/equipment	HW with midwifery competencies	Equipment	Yes
5. Perform removal of retained products of conception (e.g. manual vacuum aspiration, dilation and curettage)	B and C	Lighting	Wash hands/equipment	HW with midwifery competencies	Equipment	Yes
6. Perform assisted or instrumental vaginal delivery (e.g. vacuum extractor)	B and C	Lighting	Wash hands/equipment	HW with midwifery competencies	Equipment	Yes
		Vacuum extractor				
7. Perform neonatal resuscitation (with bag and mask)	B and C	Lighting	Wash hands/equipment	HW with midwifery competencies	Equipment	Yes
8. Perform surgery (e.g. caesarean section)	C only	Lighting	Wash hands/equipment	Team inc MW and obgyn	Equipment	Emergency referral ambulance
		Surgery equipment			Drugs	
					Supplies	
9. Perform blood transfusion	C only	Lighting	Wash hands/equipment	Team inc MW and obgyn	Equipment	Emergency referral ambulance
		Refrigerator for storage of blood			Drugs	
					Supplies	
					Blood	

Source: [15]

Poor infrastructural development in sub-Saharan africa, particularly in its rural areas has been cited as the main contributor to the poor progress made in the achievemnt of MDGs 4 and 5 [4, 17, 18, 21, 22]. Rural communities are mainly served with poor roads, inadequate health personnel and face inadequacy in the provision of essential services arising from lack of electricity and adequate water supply particularly in the lower-tier facilities [1, 18, 20, 22, 23]. These areas also experience shortages and unequal distribution of midwives, nurses and doctors, facilitating an inadequacy which puts a strain on the few health workers, overburdening and overstressing them and rendering them incapable of offering adequate and quality care [17, 18, 20, 24–27]. Poor access to affordable and clean energy and adequate water in health facilities in these areas has also been found to be a major contributor to high maternal and child morbidity and mortality in the region [28–33]. Lack of electricity makes it impossible to run cold chains that can store life-saving vaccines [32], while inadequate clean water impacts sanitation where infectious diseases may thrive and spread [27, 28, 34–36]. This situation makes timely and affordable access to the crucial maternal and child health services a challenge [18, 20, 31, 37, 38].

## Study area

**Fig 2:**
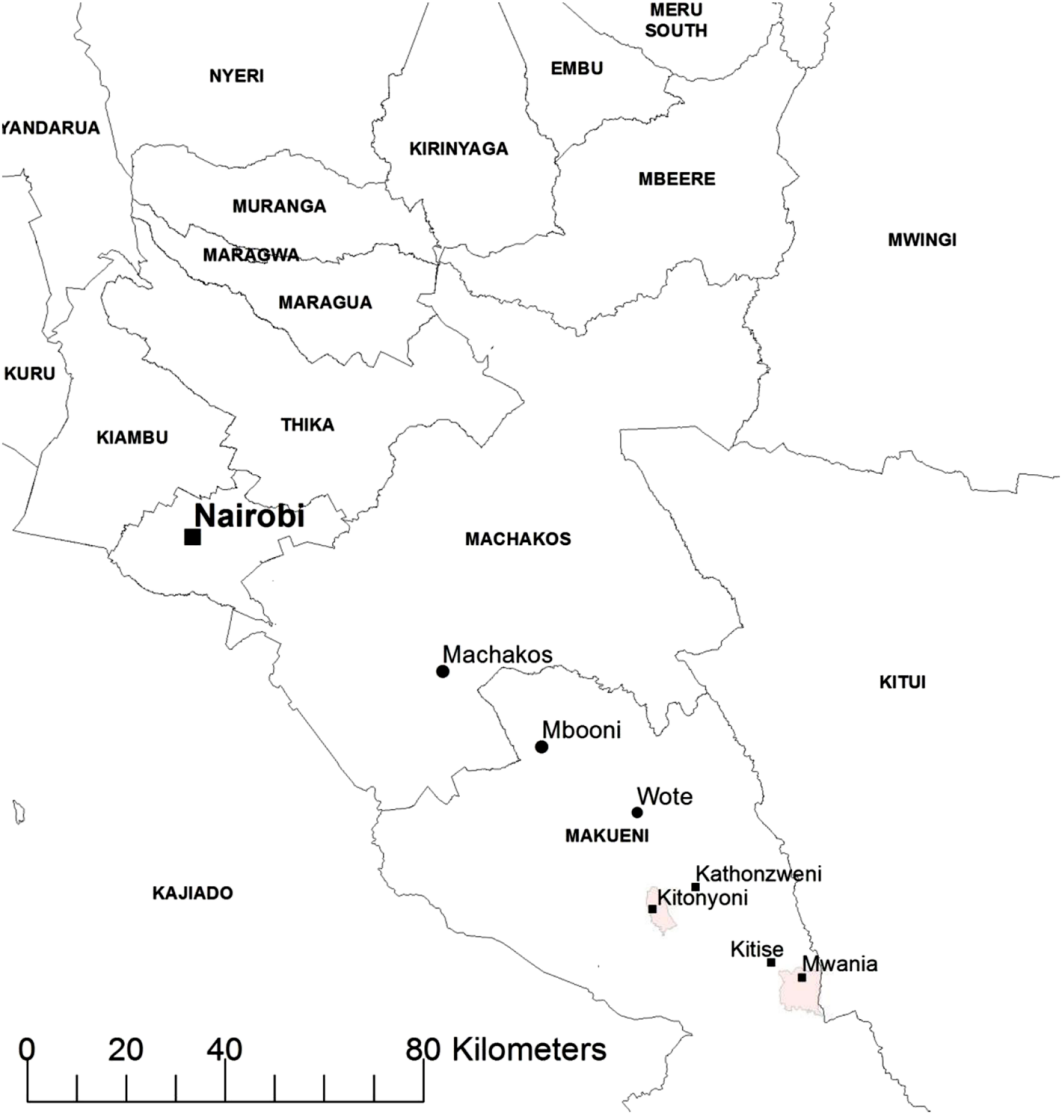
Map showing Kitonyoni and Mwania study sites

The study was conducted in the Kitonyoni and Mwania sub-locations of Makueni County, Eastern Kenya (Fig. 2). Kitonyoni which has a population of 2590 (1284 males and 1306 females) and comprises of 462 households covers an area approximately 27 square kilometres and is demarcated into 10 administrative villages. Mwania has a population of 3239 (1569 males and 1670 females) made up of 599 households and covers an area approximately 63 square kilometres and it is demarcated into 16 administrative villages. The two study areas which are about 38 km apart were chosen because they are part of a larger study assessing the impact of off-grid electricity on the wellbeing, education and health status of the rural poor in Africa. About 95 % of the roads connecting the two communities are untarred. The area is semi-arid with minimal rainfall between November and December during which the people grow maize, beans, green grams, chickpeas, cowpeas and pigeon peas for subsistence [39].

With regards to access to health care, Kenya operates a five-tier health care system. At the apex are the national referral hospitals which are teaching and research hospitals providing advanced diagnostic, therapeutic and rehabilitative care. There are two such hospitals in the country – the Kenyatta National Hospital in Nairobi and Moi Teaching and Referral Hospital in Eldoret. The nearest such facility to the study area is the Kenyatta National Hospital, located more than 140 and 170 km from Kitonyoni and Mwania, respectively (Fig. 2). The second-tier Provincial Hospitals act as referral facilities to their district hospitals and provide specialised care including intensive and specialist consultations. The Embu Provincial General Hospital is the designated Provincial Hospital serving the Makueni District, located about 165 km from Kitonyoni and 190 km from Mwania.

At the third level are district hospitals which provide integrated curative and rehabilitative care and usually have the resources to provide wide-ranging medical and surgical services including caesarean section and blood transfusion. The Makueni District Hospital located in Wote is the nearest to both study communities, located about 27 and 45 km from Kitonyoni and Mwania, respectively. The fourth-tier of health care provision is the Health Centres which provide ambulatory preventive and curative services, focusing on local needs. The nearest Heath Centres to both communities are the Kathonzweni Health Centre (located 13 km from Kitonyoni and 34 km from Mwania) and the Kitise Health Centre (located 34 km from Kitonyoni and 5 km from Mwania). Dispensaries are the lowest-tier of health care provision in Kenya. These are usually small outpatient facilities managed by Community Enrolled Nurses and supervised by a Nursing Officer from the nearest Health Centre. Kitonyoni and Mwania are each served by one dispensary. With regards to essential maternal and newborn care, the nearest EmONC facility where maternal and newborn complications are often referred is the Makueni District Hospital.

## Data collection

The data for the analysis come from qualitative interviews conducted in Kitonyoni and Mwania with the primary aim of understanding challenges faced in providing and accessing essential obstetric and newborn care services. This data collection was part of a bigger study in the two communities investigating the impact of off-grid electricity on health and wellbeing. Data were collected through FGDs with mothers and partners and key informant interviews with health care providers and community leaders. In total, 12 FGDs (6 in each site) and 4 key informant interviews (2 in each site) were conducted (Table 2). Each FGD comprised between 10 and 12 respondents selected using both stratified and purposive sampling techniques from the 10 and 16 villages in Kitonyoni and Mwania respectively. Using the E4D sampling frame of all members of the two communities, one respondent from each of the FGD categories (Table 2) was sampled from each village and formed the groups. This selection process was adopted in order to ensure that all the villages were represented in the data collected. The KII health providers were individuals in charge of the two health facilities in the two study sites while the two leaders of the two communities comprised the other KIIs. Ethical approval for the study was obtained from the University of Southampton’s Ethics Committee as well as from the Kenya Medical Research Institute’s Ethics Committee. Informed consent was obtained from participants before discussions were held. Data collection was undertaken between March and May 2011.

**Table 2:** Study respondents

Method & respondent	Age-category	No. of groups/individuals
KII Health care provider	Adult	2
KII community leader	Adult	2
FGD Women	18–24 years	2
FGD Women	25–40 years	2
FGD Women	41-59 years	2
FGD Men	18–24 years	2
FGD Men	25–40 years	2
FGD Men	41-60 years	2

The FGDs and key informant interviews were conducted using interview guides developed by the researchers. KII respondents were approached at their offices while each FGD conducted comprised participants sampled from each of the villages in the two communities in order to balance the views emerging from the service users. FGDs were conducted in the local Kamba dialect to enable respondents to freely express themselves, whilst the key informant interviews were conducted in English because the respondents could competently express themselves in English. All the interviews were audio recorded, transcribed and translated into English where necessary. The mothers interviewed were aged between 18 and 59 years, whilst the partners were between 18 and 60 years old. NVivo9 software was used for the analysis [40]. In some instances, verbatim quotations have been used to illustrate responses on relevant issues and themes.

## Results

### Livelihoods of study communities

The main economic activities in the sub-locations include subsistence agriculture, beekeeping, small scale trade and subsistence goat farming. The area is typified of acute food insecurity during the long dry spells contributing to high dependency of a large proportion of the population on government or donor food aid [41].

### Respondents’ characteristics

Table 3 gives a summary of the respondents’ personal characteristics. From this table, a majority of those responding in the study had either pre-primary or primary level of education. In addition, most of the respondents engage either in farming or in small business activities as a means of earning a livelihood, a situation that mirrors the community’s state of livelihood.

**Table 3:** Respondent characteristics

	*N*–211	
Characteristic	Male	Female
Age		
18–24 years	34	36
25–40 years	35	36
41-60 years	36	34
Educational status		
Pre–primary/Primary	100	96
Secondary+	10	5
Employment status		
Employed	28	15
Self (farming/business)	88	75
None	0	5
Marital status		
Married	94	95
Single/widowed	12	10
Parity (number of children)		
None	13	7
1–3 children	46	65
4+ children	43	37

### The state of maternal and newborn care

It emerged that health care providers and service users in Kitonyoni and Mwania are confronted with many infrastructural challenges in providing and accessing quality maternal and newborn care services. There was overwhelming consensus within and between the FGDs as well as the key informant interviews that the main challenges adversely impacting provision and access to quality maternal and newborn care include inadequate qualified health personnel, lack of adequate transportation and poor roads, lack of quality and adequate water and electricity as well as abject poverty in the communities. In each of the dispensaries that serve the two communities, there is one trained health personnel, supported by untrained health assistants (2 in Kitonyoni and 1 in Mwania). The trained health personnel in both facilities hold a Certificate in Nursing accredited as Kenya Enrolled Community Health Nurses. They are trained in the basic skills to manage normal pregnancies, childbirth and postpartum care as well as to educate and mobilise community resources to support health care provision at the local level. The facilities lack qualified doctors and highly qualified nurses to meet the challenging demand for high quality care, a situation reported to be adversely affecting provision and access to quality maternity and newborn care by both the service providers and users.

Both service providers and users reported the lack of capacity by the two health facilities to provide round-the-clock services due to inadequate staffing and lack of resources to operate at night. It was reported that the health facilities have to shut anytime the Enrolled Nurses attend meetings or when they are on annual leave. In case of the later, the facilities are sometimes shut for up to one month continually, a situation reported by service users as frustrating and inconveniencing as they are often forced to seek health care elsewhere:

“*…like now if the nurse is on leave, the hospital here is closed for one month, so we have to travel all the way to Kitise and when it is an emergency we are referred to Wote. We go through many problems when transporting a patient to Kitise or Wote. If we do not do it, the person may die.”* [Partner, FGD, 40–60 years, Mwania].

The inadequacy of health personnel affected service providers both professionally and socially as they were compelled to work extra hours to meet the demands of the community. This included working both during the day and also being called on at night or when on leave to attend to emergency cases arising in the community. It was easier for the service seekers in Kitonyoni to access services from the health worker in Kitonyoni, compared to those in Mwania, as nurse in Kitonyoni resided closer to the facility making it easier for the community to access her even at night. The service provider for Mwania however resided at Kitise, a distance of 5 km from Mwania, making it difficult for the community to access his services during out-of-office hours due to the unavailability of transport facilities.

These challenges sometimes compel service users to resort to alternative care, often from untrained Traditional Birth Attendants (TBAs). At times they opt for self-medication or decide not to seek care at all. Most women services users reported that seeking care from TBAs was a more desirable alternative because they are guaranteed the TBAs will always be available to provide care and support. However, the health personnel reported that the women sometimes encounter serious complications, which are often delayed because of dependence on TBAs. They reported that TBAs sometimes employ dangerous practices, such administration of herbal concoctions, manually changing the baby’s position in the womb which sometimes leads to antepartum haemorrhage as well as using very hot water to aid contractions, putting the life of both mother and child at risk.

Another major challenge highlighted by all those responding in this study is the lack of electricity, a situation that makes it difficult to provide round-the-clock services as well as many basic but essential maternal and newborn services. It was reported that the health facility in Kitonyoni generates lighting through the use of paraffin; there was no heating and onsite sterilisation facilities, while refrigeration was powered by Liquefied Petroleum Gas (LPG). The Mwania health facility on the other hand has no lighting facility, relies on LPG for heating, refrigeration and sterilisation of equipment. The cost of LPG was reported to be co-shared between the community and the District Health Office. However, stock outs were reported to be common due to bureaucratic protocol in releasing funds and also in cases where the community is unable to meet its share of the cost, particularly when health care fees collected from patients are inadequate.

The lack of electricity as reported by the health personnel poses a major challenge when conducting deliveries, particularly at night. They pointed out that they often have to rely on torch lights, lamps or feeble lights from mobile phones when performing deliveries. The health providers expressed their frustration of attending to birthing women at night in the captions below:

“*At night, I usually place the lamp either on a carton box like this one, or on another bed or somewhere raised. It is very challenging because I cannot keep on calling relatives of the mothers to come and assist me like with holding the lamp, because I am all alone here*” [Nurse, Key Informant Interview]

“…*sometimes I use a torch. It is very difficult to hold it and sometimes I am forced to hold the torch in the mouth as I conduct the delivery. This is because, if you have gloved yourself ready to conduct a delivery, it is difficult to hold the torch at the same time*” [Nurse, Key Informant Interview]

A recognisance of the dispensaries and discussions with the health personnel revealed that none of the nine signal functions were in place at both facilities to cater for maternal and newborn complications. The lack of electricity was cited as the main challenge to providing any of the nine signal functions. It was mentioned that although most antibiotics can be given orally, e.g. amoxicillin tablets, those that require intravenous administration including uterotonics were not available at the facilities since they are required to be stored at temperatures of between 2 and 8° Celsius to maintain their efficacy. The health facilities could not provide these services due to the lack of electricity for refrigeration. Resuscitation and assisted delivery could not be performed at the two facilities due to the lack of oxygen masks and suction machines which also require electricity to function. In addition, the health providers lack the skill to perform complex resuscitations. Instead, they resort to rudimentary and traditional techniques, as described by a health provider:

*“I do not perform resuscitation here because we do not have oxygen masks. There is no electricity here to operate these machines. I can only use the local methods…locally we make noise near the baby so that the baby can be shocked into waking up. For complex resuscitation, this is given through the umbilical cord, so if needed I usually call for help from health workers from Kathonzweni Health Centre, but this is very far…”* [Nurse, Key Informant Interview]

The magnitude of lack of apparatus to provide basic maternal and newborn care services in the facilities was further described by a service provider in the quote below:

*“…there are too many problems in this hospital, like shortage of drugs, lack of working materials e.g. delivery kits, suction machines [forceps or a ventouse suction cup] for babies who are asphyxiated [tired when being born], we do not have machines to suck the secretions, we do not have stitching kits and autoclaves to sterilise used instruments”* [Nurse, Key Informant Interview]

In addition, service providers reported that the health facilities are not able to provide regular routine services requiring refrigeration such as immunisations, where medicines are required to be stored in cold chains. Although both facilities had refrigerators, they were not in operation most of the time due to gas stock-outs. In such circumstances, vaccines are either transferred to nearest facilities with functioning refrigerators or discarded and mothers referred to other facilities for immunisation of their children. This results in feelings of frustration from service users as expressed:

“…*we are not able to immunise our children, we are told the drugs have to be put in a fridge [require refrigeration] but here there is no electricity, so they cannot keep the drugs here so we have to go to another facility…“*[Mothers, FGD, 25–39 years, Kitonyoni]

Acute water shortage particularly in the dry season was also reported by both the service providers and users as a major challenge to providing and accessing maternal and newborn care services. As indicated earlier, the main source of water for the two facilities is rainwater. It was reported that the health facilities harvest abundant rainwater during the rainy season, but storage facilities are limited thereby resulting in severe shortages during the long dry season. The dry period as reported is characterised by long trekking to water sources, reliance on poor quality water and purchasing of water at exorbitant prices to cater for the needs of their households. At the health facilities, health providers are often forced either to go without water, or use poor quality water as they are often unable to afford purifying chemicals. In addition, the scarcity of water poses serious hygiene and sanitation problems in the two facilities, especially during the provision of delivery services. It was reported that water shortages sometimes becomes so acute that it is difficult to get water for hand washing, cleaning delivery surfaces (which is a normal table that sometimes acts as a bed for examining patients) and cleaning cutting equipment, exposing mothers and newborns to infections, a frustration expressed by a service provider in the quote below:

“*After delivery, this place gets really soiled, there is no running tap water in here to clean the room, there is no water for mothers to take a shower after delivery…*” [Nurse, Key Informant Interview].

The lack of electricity and quality water also adversely impacts the recruitment and retention of qualified personnel at the health facilities. It was reported that qualified personnel are often not motivated to work in such deprived areas as the lack of electricity and water not only impacts their work at the facility but at home, they are often compelled to use alternative sources of energy and poor quality water.

### Accessing maternal and newborn referral services

The deprived state of the Kitonyoni and Mwania health facilities necessitates that pregnant and postpartum women seek referral services in better-equipped health facilities in the case of complications. Infrastructural challenges including the lack of adequate transport facilities and poor quality roads act as serious impediments to seeking referral care, often with adverse outcomes. This is compounded by poor road connectivity characterised by hills and rivers during the rainy season. The nearest referral district hospital (Makueni District Hospital in Wote) although located only 27 km from Kitonyoni and 45 km from Mwania, it was reported that on average it takes about three hours to travel to the facility due to the poor nature of the connecting roads, particularly during the rainy seasons. Due to the fact that there is no motorised ambulance serving the dispensaries, patients often use a combination of commercial motorbike, mini-bus and taxi to make this journey. It emerged that mini-bus operations were infrequent (operates only twice during the day). Taxi services were said to be very expensive (between Ksh. 2000 and Ksh. 4000; equivalent of $23-$46 for a one way journey) and often has to be called from Wote where the district hospital is located. In such circumstances, the woman or her family were not only faced with delays in getting to the hospital, but were also often made to pay for the double journey. As one young mother described:

*“…like now, there are transport difficulties, in case there is a need for referral, we do not have a vehicle here, we have to call for taxi from Wote and it is very far and expensive”* [Mother, FGD, 18–24 years, Mwania]

The health providers bemoaned that the difficulties of seeking referral services sometimes lead to fatalities or near-misses, particularly where mothers delay in seeking hospital care, as indicated in the quote below:

“*When I first started working here, there was a woman who had been in labour for 2 ½ days without delivering… we decided to refer her, but there was no transport and the family did not have money for taxi. I decided to use my money to take a taxi because there was no matatu [public transport] operating. There was so much rain and we got there too late. The woman died shortly after delivery, but the baby survived*” [Nurse, Key Informant Interview]

Such occurrences do have a devastating impact on the family, as one husband who recently lost a wife iterated sorrowfully:

“*When I lost my wife, I was left with the baby and this is very hard life because I have to be both the father and mother. Now the baby is bigger [grown] but things were difficult in the beginning because I had to buy milk for the baby and I do not have a job*” [Partner, FGD, 18–24 years, Mwania].

The respondents also recounted that the poor road quality and lack of adequate transportation further lead to hikes in transportation prices, particularly at night and during the rainy season. This is further made worse if the health of the woman is so bad that she cannot walk and a commercial motorbike has to be hired to take her to the main road. The patient’s condition as was reported by mothers is often exacerbated by the discomfort of the motorbikes due to the rough terrain. Considering that the cost of taxi from the two study communities to Wote ranges between 2000 and 4000 Kenyan Shillings ($23-$46), very few are able to afford. This is reinforced by a woman respondent in this quote:

“*From here to Wote we have to pay Ksh. 2000 for taxi just to take you there. We cannot afford this, so if there is no matatu [public transport], we just have to resort to other means [alternative treatment]*” [Mother, FGD, 25–39 years, Kitonyoni].

The health providers iterated that one of the major complications they face which requires speedy referral care is severe bleeding. They noted that a woman experiencing severe bleeding could die within two hours if not attended to, even if she is in good health. They reported that the health facilities do not have blood transfusion facilities, neither are they able to store oxytocin (as they are most effective when stored in cold chains, given the aridity of the area) to administer after childbirth to effectively reduce the risk of bleeding. It was reported that this often leads to fatalities and near-misses, as even in cases where women are referred to the district hospital, they are less likely to get to the referral facility within two hours due to the poor roads, lack of transport and exorbitant transport costs.

Poor transport facilities ultimately impacts not just referrals, but also access to the necessary hospital supplies, often resulting in stock-outs of essential medicines. In such situations, the health care providers are not able to offer essential services. Sometime they have to access supplies themselves using commercial motorbikes, in which case the dispensary has to shut for the time the providers are away from the facilities.

### Community perceptions of transport infrastructure as a barrier to accessing care

It was evident from the study that financial cost, both health and non-health expenditures (e.g. consultation and admission fees, cost of medicines and transportation) are major barriers to accessing maternal and newborn care services in the study communities. Maternal and newborn care services expenditure as reported by respondents do exacerbate poverty not only through the expenditure incurred but also through loan negotiations to solicit for funds to pay for services. In some cases women forgo care to avoid the financial costs. In this regard, it was reported that only few relatively well-to-do households in the community are able to access health care from better-equipped facilities located in the district capital (Wote). Frequent drug stock-outs in the health facilities require that pregnant and postpartum women often have to purchase prescribed medication, a situation that is often not affordable for the poor in the community. One partner expressed his helplessness in this quote:

“…*you are prescribed drugs to go and buy…With my meagre income I will struggle to pay for the services and sometimes one is forced to go back home with my wife without the treatment”* [Partner FGD, 39–60 years Kitonyoni].

## Discussions

The aim of this study was to examine the infrastructural challenges of providing and accessing basic maternal and newborn care services in the remote rural communities of Kitonyoni and Mwania in Eastern Kenya. It is widely acknowledged that accelerating progress to the UN maternal and newborn health targets (MDGs 4 and 5) in low and middle income countries requires that barriers limiting access to essential and quality maternal and newborn care services are identified and addressed at all levels of the health system. The efforts and commitments to address this issue has focused primarily on providing access to life-saving interventions based on the nine signal functions proposed by the World Health Organisation [16]. The lowest-tier of health facilities which serve remote rural communities often lack the prerequisites to function at the very basic level of providing routine services. The basic needs of these facilities are not captured in the nine signal functions, thus it is imperative to identify and understand the challenges of providing and accessing basic maternal and newborn care services in these communities so as to alert policy makers and programme managers to their needs.

The findings from this study confirm that the challenges of providing basic and routine maternal and newborn care services are not restricted to those described in the nine signal functions. These challenges include personnel and infrastructural shortcomings such as poor quality roads and lack of transportation, lack of electricity and quality water as well as poverty, often exacerbated by the cost of accessing maternal and newborn care services. Although the factors identified in this study concur with others studies in sub-Saharan Africa [42–45], it has provided further insights into the challenges of providing and accessing basic maternal and newborn services in rural communities, which are often not covered in national level intervention strategies and programme actions.

The findings from this study clearly show that the needs of these communities are not only prerequisites for proving basic or comprehensive EmONC services, but also for providing routine services in lower-tier health facilities. The findings shows that these facilities are not capable of providing round-the-clock services as well as basic but essential maternal and newborn services including routine immunisations and ensuring hygienic delivery. This was attributed to inadequate skilled personnel and infrastructural challenges including the lack of energy to power cold chains and sterilise equipment. The unavailability of reliable water supply for performing clean births expose women and newborns to tetanus and sepsis, conditions highly associated with maternal and neonatal mortality [32]. Although the World Health Organisation recommends that maternal and newborn services should be available round the clock, it is evident that service provisions in these communities are contrary to these recommendations [23].

The findings further suggest that the unavailability of round-the-clock services compel women to seek care from unskilled providers such as TBAs. Although evidence in some studies show that TBAs are often crucial in filling the gap that the unavailability of qualified health providers can fill, some practices adopted by some of them sometimes lead to adverse outcomes [45]. The lack of electricity and quality water supply also affects recruitment and retention of qualified health personnel in the communities. Yet, the United Nations recognises that providing basic necessities such as clean water and reliable electricity can attract qualified health personnel to rural areas thereby improving access to crucial maternal and child health services [46].

The poor state of maternal and newborn care services in the study communities necessitates that women often seek referral services from the district hospital. The poor nature of the connecting roads and lack of adequate and reliable transportation are major impediments to accessing timely referral care. This is made worse during the rainy seasons and also at nights as transport providers hike their fares beyond what most members of the community can afford. The unavailability of ambulance services requires that pregnant women are conveyed by inappropriate transport including commercial motorbikes and taxis, often exacerbating their already poor health. Delays in getting pregnant women to the referral facility sometimes lead to fatalities and near-misses. The poor nature of roads and transport facilities also affects hospital supplies, often resulting in stock-outs of essential medicines.

These challenges are compounded by the level of poverty experienced in the communities and the high expenditure involved in seeking health care. This is often aggravated by loan negotiations that households have to undertake to solicit for funds to care for pregnant women and newborns. The challenges of providing and accessing maternal and newborn care services in these rural communities are interrelated such that they effect each other or act together to increase the vulnerability of an already poor and vulnerable community. This finding concur with other studies in rural communities of sub-Saharan Africa which reported that the poor continue to pay more for health-related services due to poor infrastructural development and under-resourced health facilities [47].

## Conclusions

There is clear evidence that progress towards the fourth and fifth MDGs has been slow in most countries of sub-Saharan Africa. In some cases the progress has either stalled or is retrogressing as is the case of Kenya [2, 23]. Despite the recent spotlight on increasing urbanization, majority of Kenya’s population resides in rural areas and United Nations projections show that by 2025, a majority of the population (53 %) will still reside in rural Kenya [48] indicating the need to focus on improving the health of this segment of the population.

This study has shown that in rural communities where maternal and newborn deaths remain unacceptably high, the lack of the nine signal functions are not the only challenges to the low-tier health facilities providing services in these communities. Prerequisites such as reliable electricity, quality water and road networks, appropriate transportation facilities and adequate qualified health personnel are equally essential for providing basic routine services which these facilities are designated to provide. Thus, removing barriers to maternal and newborn care should not only focus on the provision of the nine signal functions and fee exemptions but also on improvement of infrastructure to facilitate provision and access to care as well as attracting skilled health personnel to rural areas. Improving infrastructure in rural areas also has the potential to reduce out-of-pocket costs for seeking skilled maternal and newborn care.
